# Aerobic Exercise Training in Post-Polio Syndrome: Process Evaluation of a Randomized Controlled Trial

**DOI:** 10.1371/journal.pone.0159280

**Published:** 2016-07-15

**Authors:** Eric L. Voorn, Fieke S. Koopman, Merel A. Brehm, Anita Beelen, Arnold de Haan, Karin H. L. Gerrits, Frans Nollet

**Affiliations:** 1 Department of Rehabilitation, Academic Medical Center, University of Amsterdam, Amsterdam, The Netherlands; 2 Department of Human Movement Sciences, Faculty of Behavioural and Movement Sciences, Vrije Universiteit Amsterdam, MOVE Research Institute Amsterdam, Amsterdam, The Netherlands; IRCCS E. Medea, ITALY

## Abstract

**Objective:**

To explore reasons for the lack of efficacy of a high intensity aerobic exercise program in post-polio syndrome (PPS) on cardiorespiratory fitness by evaluating adherence to the training program and effects on muscle function.

**Design:**

A process evaluation using data from an RCT.

**Patients:**

Forty-four severely fatigued individuals with PPS were randomized to exercise therapy (n = 22) or usual care (n = 22).

**Methods:**

Participants in the exercise group were instructed to exercise 3 times weekly for 4 months on a bicycle ergometer (60–70% heart rate reserve).

**Results:**

The attendance rate was high (median 89%). None of the participants trained within the target heart rate range during >75% of the designated time. Instead, participants exercised at lower intensities, though still around the anaerobic threshold (AT) most of the time. Muscle function did not improve in the exercise group.

**Conclusion:**

Our results suggest that severely fatigued individuals with PPS cannot adhere to a high intensity aerobic exercise program on a cycle ergometer. Despite exercise intensities around the AT, lower extremity muscle function nor cardiorespiratory fitness improved. Improving the aerobic capacity in PPS is difficult through exercise primarily focusing on the lower extremities, and may require a more individualized approach, including the use of other large muscle groups instead.

**Trial Registration:**

Netherlands National Trial Register NTR1371

## Introduction

Individuals with post-polio syndrome (PPS) generally report fatigue as their main problem [1,2]. This fatigue is known to be a multidimensional symptom and consists, besides cognitive and psychological components also of a physical component [3]. One of the factors responsible for physical fatigue may be a reduced aerobic capacity resulting from a lower physical activity level [4,5]. Recently, we reported the results of a randomized controlled trial (RCT) in PPS that failed to show improvements in fatigue after a 4-month exercise therapy (ET) intervention with a home-based high intensity aerobic exercise program [6].

In the RCT we also observed no change in cardiorespiratory fitness, assessed from the submaximal heart rate response [6], which contradicts findings of several previous studies [7,8]. Clarifying the reasons for the lack of efficacy of our exercise program will provide insight in the potential role of aerobic training and in optimal training methods for alleviating fatigue symptoms in PPS.

One possible explanation for the lack of efficacy is that individuals did not adhere to the program. During the program, designated training intensity was gradually increased from 60% heart rate reserve (HRR) to 70% HRR, which is in accordance with the American College of Sports Medicine guidelines for aerobic training in persons with chronic diseases [9]. In addition, training duration was increased from 28 to 38 minutes per session. Although it has been reported that individuals with PPS can tolerate such high intensity programs [7,8,10–13], most of these studies provided incomplete or no insight in the training intensity and duration actually achieved. It is therefore currently unclear whether individuals with PPS can adhere to an aerobic training program based on these guidelines.

Also the optimal training dose (in terms of intensity and duration), its relationship with the subsequent training response and the mechanisms of improvement are currently still unknown in PPS. It is well known that regular aerobic training induces both central (i.e. cardiorespiratory) and peripheral (i.e. muscular) adaptations [14]. Furthermore, improvement of cardiorespiratory fitness requires involvement of large muscle groups to impose an adequate stimulus for adaptations [15]. Possibly, our exercise program did result in muscular adaptations, which, due to the reduced muscle mass of the lower extremities, did not lead to an increased cardiorespiratory fitness. On the other hand, if no muscular adaptations occurred, this indicates that the training dose was apparently insufficient to induce a positive training response.

In the current study we present the results of a process evaluation of the ET intervention as given in our RCT. We investigated the following research questions: (1) Do individuals with PPS adhere to a 4-month high intensity home-based aerobic training? (2) Does a high intensity home-based aerobic training result in improved lower extremity muscular function? (3) To what extent does actual training dose explain the variance in training response?

## Methods

### Design

The data used in the present study comes from the multicenter RCT on the efficacy of ET and cognitive behavioral therapy on reducing fatigue, and improving activities and quality of life in patients with PPS (S1 protocol). The RCT was registered at the Netherlands National Trial Register (NTR1371). Two previous publications describe the study design [16] and main results [6] of the trial. In the present study, we compared the group allocated to ET with the usual care (UC) group. Outcomes in both groups were assessed at baseline (pre-treatment) and after 4 months (post-treatment), by two assessors who were blinded for treatment allocation.

### Participants

Participants were recruited from 7 hospitals and rehabilitation centers throughout the Netherlands. Participants were initially screened by a physician to check the in- and exclusion criteria. The most important inclusion criteria were: diagnosis of PPS according to the criteria as published by the March of Dimes [1]; severe perceived fatigue (subscale fatigue severity of the Checklist Individual Strength (CIS20-F) ≥35) [17]; and ability to cycle on a cycle ergometer against a load of ≥ 25 Watts. A more detailed description of the in- and exclusion criteria is available elsewhere [16]. Our study protocol was approved by the Medical Ethics Committee of the Academic Medical Center in Amsterdam, and all participating centers granted approval to participate. Written informed consent was obtained from all participants.

### Interventions

#### Usual care

The participants in the UC and ET group all received UC. UC for PPS could include the use of assistive devices and/or orthoses, physical therapy, and medication use. Participants were not restricted in their activities.

#### Exercise therapy

ET lasted 4 months and consisted of (1) a home-based aerobic training program on a bicycle ergometer 3 times weekly and a (2) supervised group training containing muscle strengthening and functional exercises once a week.

1. Participants were supplied with a bicycle ergometer (Kettler X7, Germany) and logbook with training instructions at their home. In the logbook, participants documented the number and duration of treatment sessions, their perceived exertion on the Borg Scale (range 6–20) [18] and possible complaints during or after the training session. During training, the heart rate (HR) was continuously monitored and stored (Polar RS400, Polar Electro Nederland, Almere, The Netherlands). Training intensity was gradually increased from 60% to 70% HRR, and the training duration was gradually increased from 28 to 38 minutes per session. For participants using beta-blockers training intensity prescription was based on the Borg Scale (12 to 13). Sessions were divided into prescribed exercise bouts (increasing from 2 to 13 minutes), interspersed with short rest periods of unloaded cycling. Actual training intensity of the sessions was monitored weekly by one of the therapists by reading the heart rate monitors and checking the perceived exertion scores and complaints documented in the logbooks and adjusted based on tolerance.
2. The supervised group training consisted of individually tailored strengthening exercises and functional exercises in 1-hour group sessions. Only muscle groups with a score ≥3 according to the Medical Research Council (MRC) scale [19] were selected for the strengthening exercises. Functional exercises aimed to improve the interplay of cognitive, perceptual, and motor functions.

### Outcome measures

#### Adherence

The attendance rate of ET was assessed by recording the fraction of home-based training sessions as recorded in the participants’ logbooks. For adherence, we used the data from the heart rate monitors to establish the total time that participants trained within their prescribed target HR range (60% to 70%HRR). Participants were considered adherent if they exercised >75% of the possible time within their prescribed target HR range.

In addition, we established the total time that participants trained at or above the HR corresponding to their anaerobic threshold (AT). This is of interest because the AT is often used to target training intensity [20–23]. We recently demonstrated that the AT occurs below 60%HRR in most individuals with PPS [24]. Therefore we also established whether participants were capable of exercising at or above their AT. We considered this to be the case if participants trained >75% of the possible time at or above their AT. The AT was determined from the pre-treatment submaximal incremental exercise test using the V-slope method [25].

#### Muscle function

For muscle function, we assessed both muscle endurance and strength. As a measure of muscle endurance we determined the fatigue resistance of the knee extensor muscles (at 60° knee flexion) by a series of intermittent electrically evoked isometric contractions (150 contractions of 1 second duration and 1 second of rest in between) with the use of a fixed dynamometer (Biodex System 3, New York, USA). Fatigue resistance was defined as the percentage torque remaining during the last minute of the protocol [26]. Measurements were performed on the weakest leg, unless during manual muscle testing grading of knee extension strength was <3, according to the MRC scale [19]. An extensive description of the protocol is available elsewhere [16,26].

For muscle strength we measured the maximal voluntary torque (MVT) of the knee extensor muscles isokinetically between 90° and 30° knee flexion at a velocity of 60°/sec using a fixed dynamometer. The MVT of each leg was measured separately and we included the best of 3 maximal efforts in the analysis. If the MRC-score was <3, strength measurements were not performed. Data for isokinetic strength measurements are presented both for the strongest leg and the weakest leg.

#### Cardiorespiratory fitness

The previously reported results, indicating no change in the cardiorespiratory fitness following training, solely considered the submaximal HR response [6]. However, other indices of HR and gas exchange outcomes, as well as values of perceived exertion could also reveal possible cardiorespiratory adaptations.

From the submaximal incremental cycle ergometry tests [16], we assessed changes in resting HR, oxygen consumption at the AT, submaximal oxygen consumption (VO_2submax_), submaximal respiratory exchange ratio (RER_submax_), and submaximal ratings of perceived exertion (RPE_submax_). VO_2submax_, RER_submax_ and RPE_submax_ were assessed at the highest similar submaximal workload that was achieved both during the pre- and post-treatment assessment.

### Data analysis

Descriptive statistics were used to characterize the sample. For normally distributed data, we used the paired-samples *t* test to test differences within the groups and the Student *t* test to test differences between groups; in case of non-normally distributed data, the Wilcoxon signed-rank test and Mann-Whitney *U* test were used.

Using a linear regression model, we determined R-squared (R^2^), representing the extent to which the actually achieved training dose explained the variance in submaximal heart rate change (ΔHR_submax_). Session training doses (calculated as the duration of the session multiplied by the mean %HRR for that session) were summated for each participant to obtain the total actual training dose [27]. We visualized the relationship in a plot.

An alpha level of 0.05 was used for all tests of significance. Statistical analyses were performed with the SPSS statistical software package (version 20.0.0.1, IBM Company, Armonk, NY, USA).

## Results

**Fig 1:**
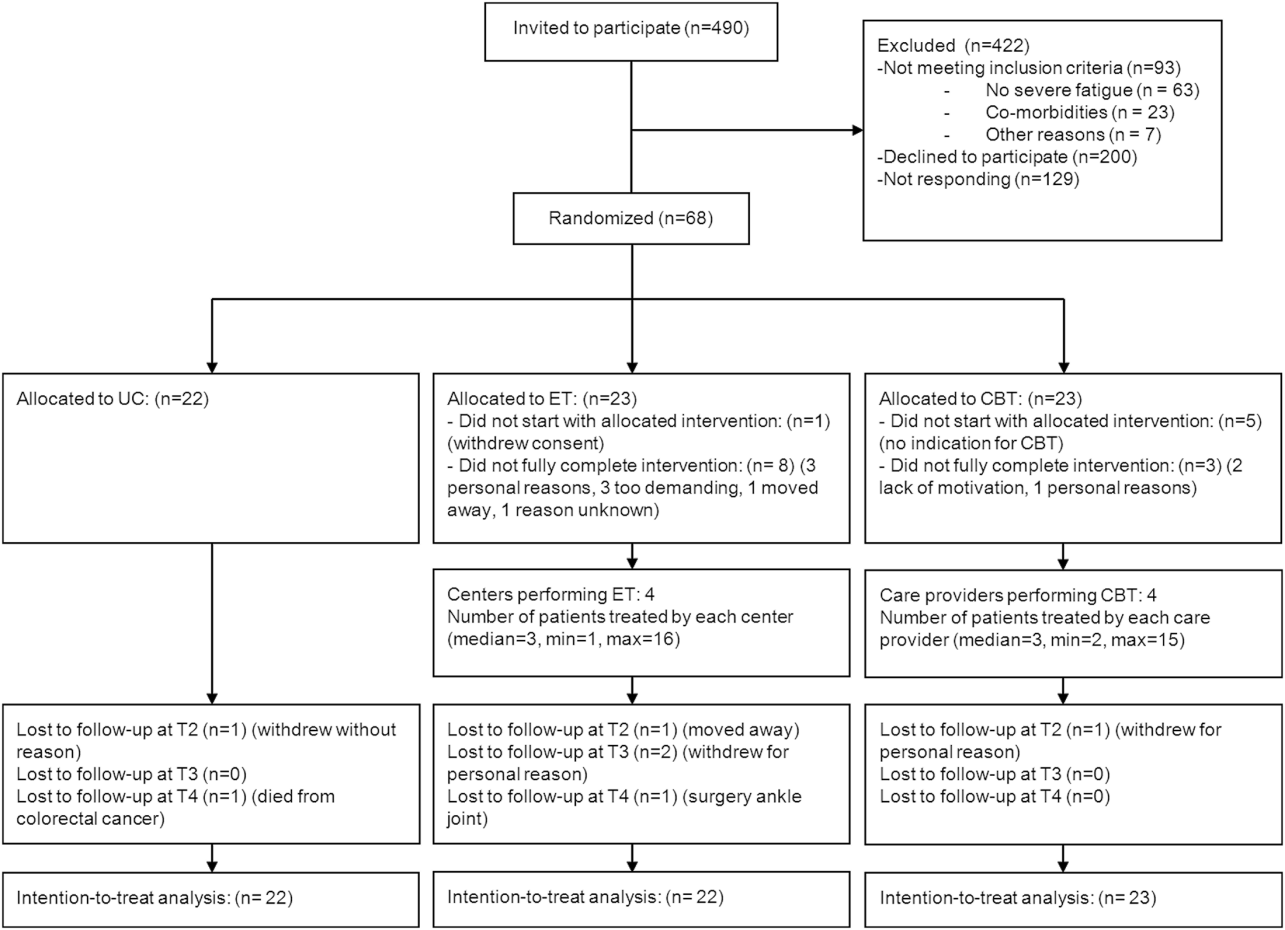
Flow diagram. Abbreviations: ET = exercise therapy; CBT = cognitive behavioral therapy.

From June 2009 to September 2012, 68 patients were enrolled in the RCT (Fig 1). In total 44 participants were included in the analyses for this study (22 in UC and 22 in ET). Table 1 shows the characteristics of participants in the UC and ET group.

**Table 1:** Baseline characteristics of participants.

	ET (N = 22)	UC (N = 22)
**Demographic data**		
Age (yr), mean (SD)	60.1 (7.4)	56.7 (8.9)
Gender, n female (%)	11 (50%)	13 (59%)
Body mass index (kg/m^2^), mean (SD)	26.5 (3.4)	25.6 (4.3)
**Polio characteristics**		
Age at acute polio (yr), median (range)	3 (1–40)	2 (0–16)
Time since new symptoms (yr), mean (SD)	13.2 (7.7)	14.6 (9.5)
Present walking distance, n(%)^a^		
Around the house	9 (41%)	6 (27%)
Seldom further than 1 km	8 (36%)	11 (50%)
Regularly further than 1 km	5 (23%)	5 (23%)
Manual muscle testing sum score legs (0 to 80), median (range)^b^	67.8 (41.8–80.0)	69.6 (43.0–80.0)
Manual muscle testing sum score arms (0 to 80), median (range)^c^	50.0 (32.0–50.0)	50.0 (16.8–50.0)

Abbreviations: ET, exercise therapy; UC, usual care; SD, standard deviation.

^a^ Walking distance was classified into 3 categories: around the house, seldom further than 1 km, and regularly further than 1 km.

^b^ Sum score for muscle strength of the legs was calculated by adding 16 muscle groups. Each muscle group had a score between 0 and 5, sum score ranged from 0 to 80 [19].

^c^ Sum score for muscle strength of the arms was calculated by adding 10 muscle groups. Each muscle group had a score between 0 and 5, sum score ranged from 0 to 50 [19].

### Adherence

**Fig 2:**
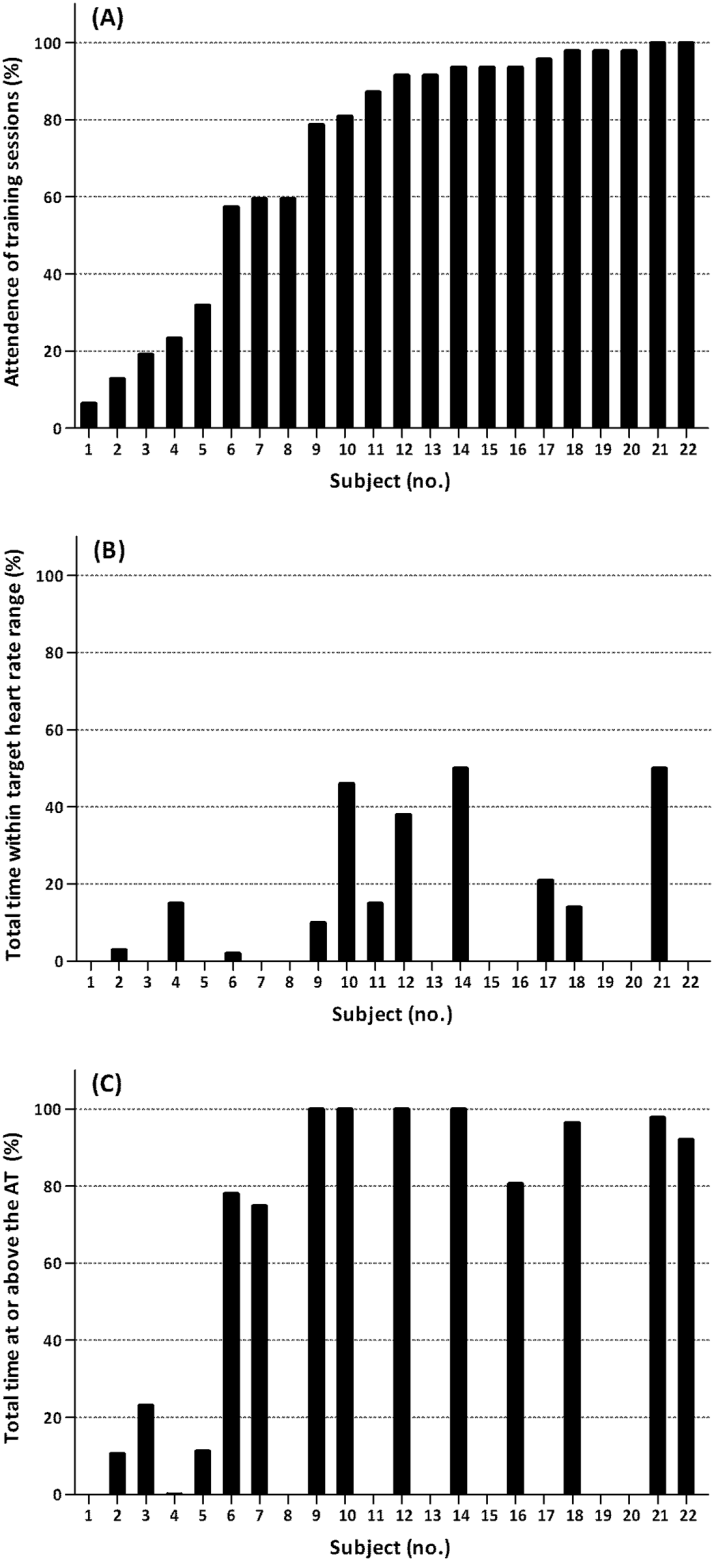
Individual attendance rates (A), the total time within the target heart rate range (B), and the total time at or above the heart rate corresponding to the anaerobic threshold (C) for participants in the exercise therapy group. Missing data in panel B: subjects 3,16 and 22 used beta blockers; heart rate data was incomplete in subjects 1,13,15,19 and 20. Missing data in panel C: the anaerobic threshold could not be identified in subjects 8,11 and 17; heart rate data was incomplete in subjects 1, 13, 15, 19 and 20. Abbreviations: AT = anaerobic threshold.

Fig 2A shows the attendance rate for the aerobic exercise sessions at home (median 89%, of 47 possible sessions). Fourteen participants (64%) attended >75% of the possible sessions. Five participants (23%) did not complete the aerobic exercise program (e.g. followed no more training sessions during the last 8 weeks of the program). The 3 most frequently reported reasons for missing a training session were fatigue, illness and muscle pain.

**Fig 3:**
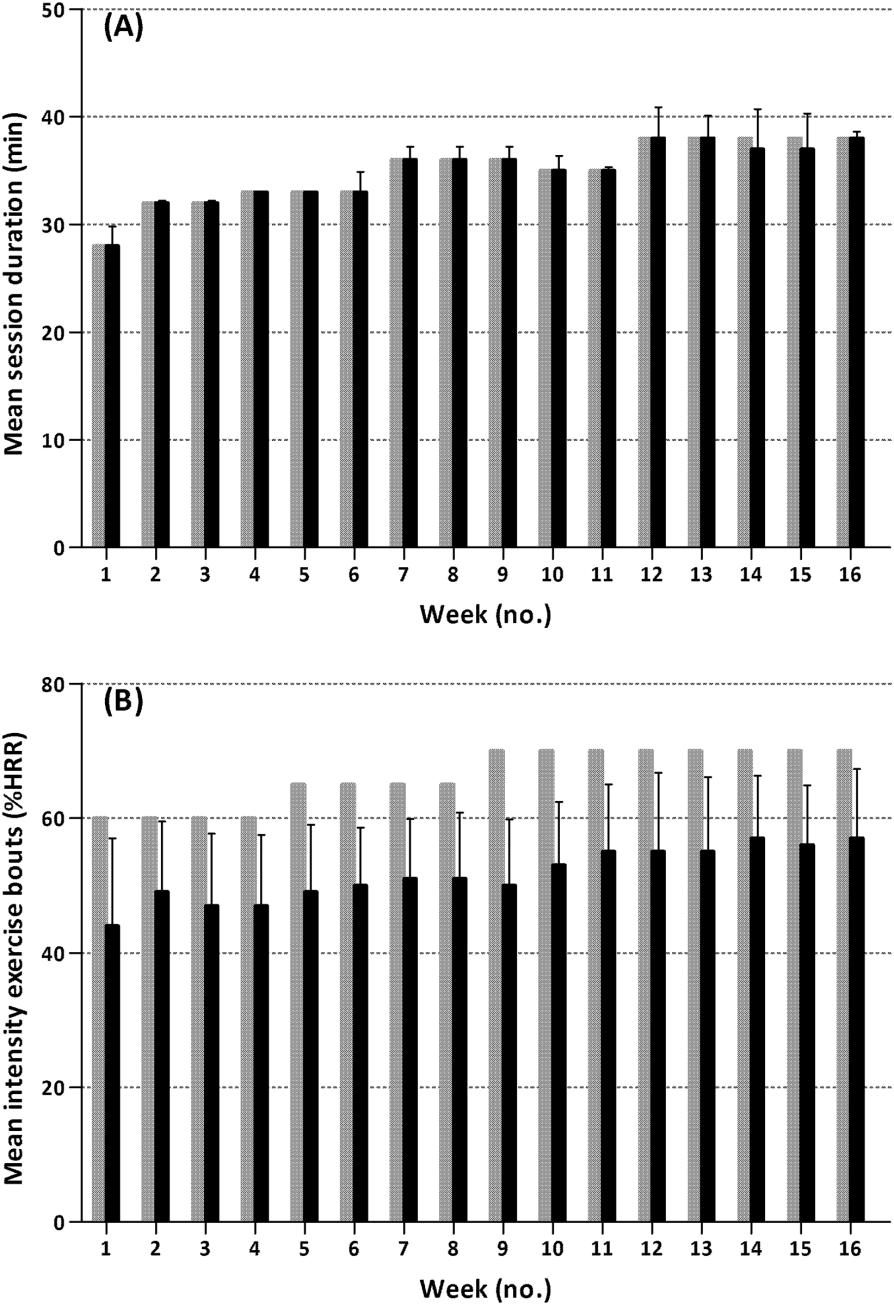
Group mean values (SD) for training duration achieved during the training sessions (A) and intensity sustained during the exercise bouts (B) for each week of the program. Grey bars indicate designated duration/intensity; black bars indicate the actually achieved duration/intensity. Abbreviations: HRR = heart rate reserve.

Three participants (14%) used beta-blockers. In addition, due to technical problems, HR data was incomplete in 5 other participants (23%). HR data in the remaining 14 participants (64%) showed that nobody trained within their designated target HR range >75% of the possible time (Fig 2B). Group mean values for the mean duration achieved during the sessions for each week of the program are presented in Fig 3A, illustrating that training duration increased in accordance with the protocol. Fig 3B shows the group mean values for the mean %HRR achieved during the exercise bouts for each training week. There was a pattern of increasing intensity throughout the entire training program, but in all except two participants, it remained below designated intensities.

We identified the AT in 18 participants (82%) of whom 14 (64%) had complete training HR data. Most of these participants (71%) trained >75% of the possible time at or above the HR corresponding to their AT (Fig 2C). Participants who attended >75% of possible sessions, all trained >75% of the time at or above their AT.

**Fig 4:**
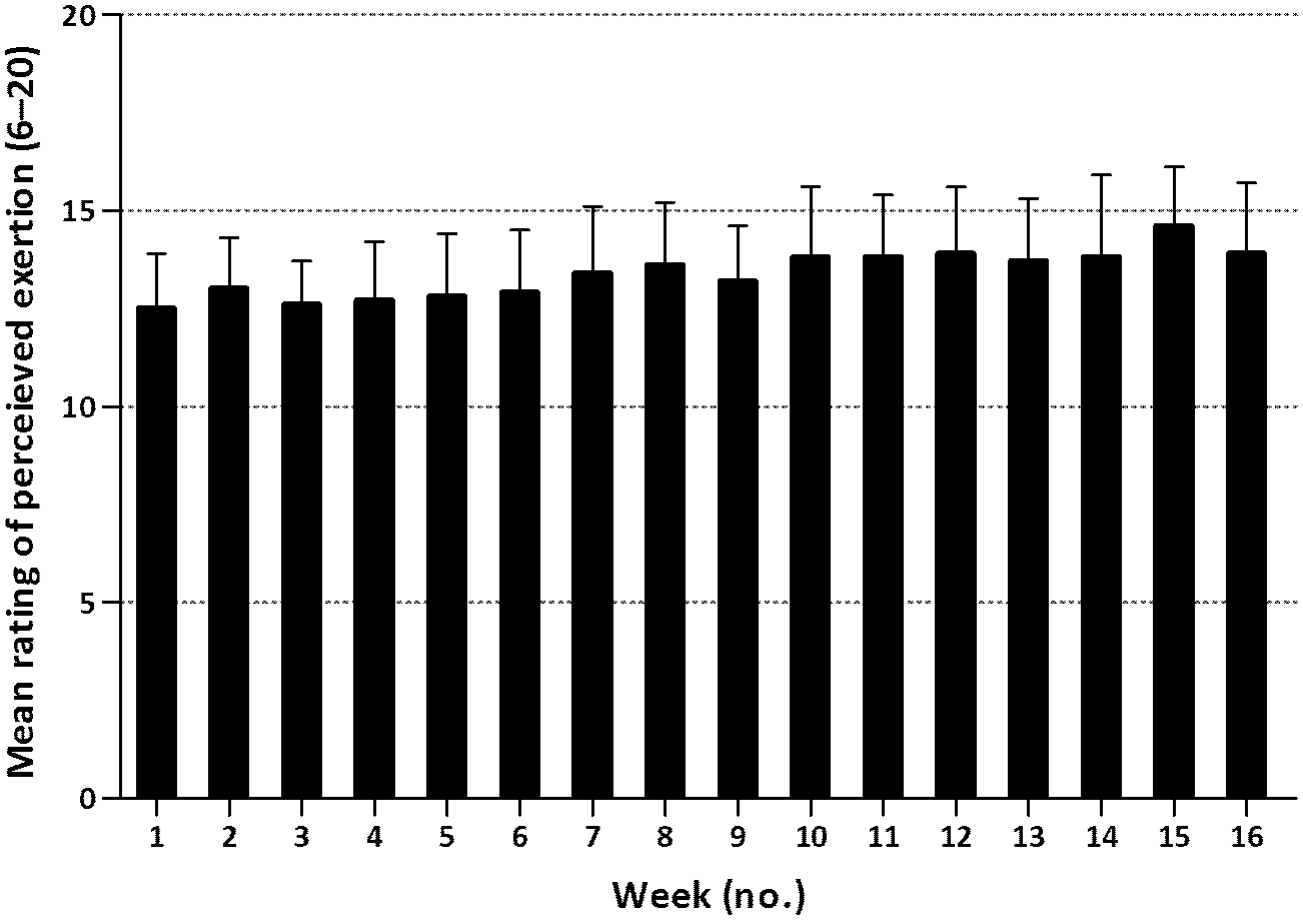
Group mean values (SD) for the perceived exertion of training sessions for each week of the training program.

Ratings of perceived exertion during the training sessions were incomplete (data in <75% of the sessions) in 8 participants (36%). In the remaining 14 participants (64%), the perceived exertion showed an increasing pattern throughout the entire training period (Fig 4). The percentage of participants who rated at least half of the weekly training sessions as 12 or higher on the Borg Scale, increased from 71%, to 91%, to 100% in the 1^st^, 8^th^ and 16^th^ week of the program, respectively.

### Muscle function and cardiorespiratory fitness

Table 2 shows the outcomes pre- and post-treatment, together with the difference in change scores between ET and UC. We found no significant effects of ET on muscle endurance (1.6%, 95% CI -10.6 to 13.7) and muscle strength (strongest leg 2.0Nm, 95% CI: -10.2 to 14.2; weakest leg 0.7Nm, 95% CI: -15.0 to 16.5) compared to UC. Furthermore, we found no changes in these muscle function outcomes within both groups, and there were also no significant differences within and between the groups for any of the cardiorespiratory fitness measures.

**Table 2:** Outcome measures pre- and post-treatment in the ET and UC group.

	ET			UC			ET vs UC
	n	Pre-treatment	Post-treatment	n	Pre-treatment	Post-treatment	Mean difference in change scores (95% CI)
**Muscle endurance and strength**							
Fatigue resistance (*% remaining torque*), mean (SD)	3	46.3 (2.5)	53.7 (6.5)	4	40.3 (2.8)	46.0 (5.9)	1.6 (-10.6 to 13.7)
MVT strongest leg (*Nm*), mean (SD)	16	100.3 (41.6)	105.1 (39.1)	20	81.9 (52.8)	84.6 (20.3)	2.0 (-10.2 to 14.2)
MVT weakest leg (*Nm*), mean (SD)	11	76.3 (40.7)	79.3 (45.0)	14	62.7 (35.7)	65.0 (34.7)	0.7 (-15.0 to 16.5)
**Cardiorespiratory fitness**							
Submaximal HR (*bpm*), mean (SD)	18	121.0 (14.1)	119.0 (12.0)	21	119.1 (19.1)	117.3 (19.4)	-0.3 (-7.8 to 7.2)
Resting HR (*bpm*), mean (SD)	18	74.2 (8.0)	77.1 (13.2)	21	73.0 (11.3)	74.5 (11.5)	1.4 (-4.5 to 7.3)
AT (*mL/min/kg*), mean (SD)	13	15.5 (4.4)	16.3 (4.3)	11	15.4 (2.4)	14.5 (2.4)	1.7 (-1.6 to 4.9)
Submaximal VO_2_ (*mL/min/kg*), mean (SD)	19	17.1 (4.6)	17.0 (4.7)	21	15.7 (4.5)	15.6 (4.9)	0.1 (-1.0 to 1.2)
Submaximal RER (*ratio*), mean (SD)	19	0.94 (0.09)	0.94 (0.06)	21	0.95 (0.08)	0.97 (0.08)	-0.02 (-0.06 to 0.02)
Submaximal RPE (*6 to 20*), mean (SD)	19	13.5 (2.0)	12.9 (2.0)	21	13.5 (2.4)	13.0 (2.1)	-0.1 (-1.5 to 1.3)

Abbreviations: ET, exercise therapy; UC, usual care; CI, confidence interval; MVT, maximal voluntary torque; HR, heart rate; AT, anaerobic threshold; VO2, oxygen consumption; RER, respiratory exchange ratio; RPE, rating of perceived exertion.

### Explained variance training dose

**Fig 5:**
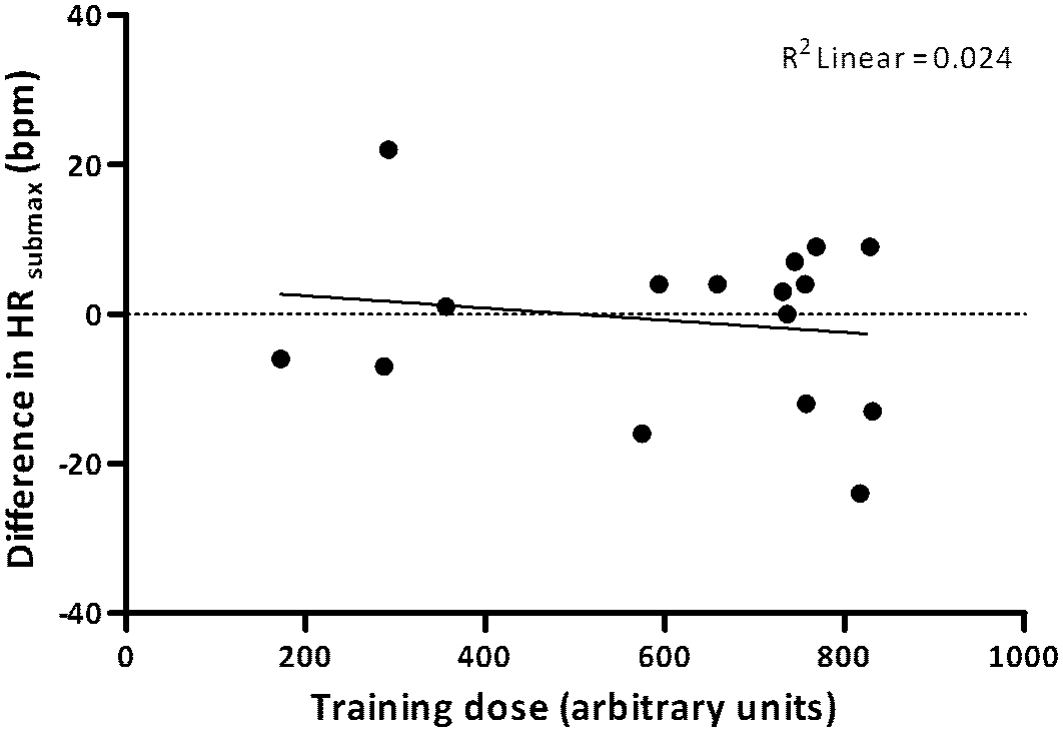
Relationship between submaximal heart rate change (ΔHRsubmax) and total actual training dose.

Fig 5 shows that training dose was not associated with the ΔHR_submax_ (R^2^ = 0.024, *P* = 0.57).

## Discussion

Results from our study show that, despite the high attendance rate severely fatigued individuals with PPS did not adhere to a 4-month high intensity aerobic exercise training. Participants instead exercised at or slightly above their AT most of the training period, but neither muscle function nor cardiorespiratory fitness improved. Our findings confirm that the training program is ineffective in increasing the aerobic capacity of individuals with PPS.

Even though participants attended most of the training sessions, our results demonstrate that only a few people exercised at high intensities (>60%HRR) for prolonged periods of time. This is surprising, given earlier reports, which showed that high intensity training programs are well tolerated by individuals with PPS [7,8,10–13]. From these reports, only two described the duration and intensity actually achieved [8,12]. Contrary to our study, participants in the 4-month aerobic exercise program of Jones and colleagues achieved a mean HRR of 69.2% [12]. The higher %HHR reported in the Jones study may have resulted from excluding the HR data of participants that withdrew the study (n = 6), and also the seemingly better exercise capacity and lower age of participants compared to our study may have contributed. Furthermore, even though little information was provided about individual variation, the authors acknowledged that, in some participants, intensity had to be adjusted downward. Modifications in exercise intensity were also required in the study by Kriz and colleagues [11], who showed that the mean HR during their arm ergometry training program represented only 50%HRR, while target intensity was set at 70%–75%HRR, a pattern similar to our study. Even though factors such as the lack of motivation and boredom cannot be ruled out, it may be argued that, for most individuals with PPS, high exercise intensities are too exhausting to sustain for prolonged periods, possibly especially in severely fatigued individuals.

While high intensities were not sustained, participants exercised at or above their AT during most of the training period. Participants rated most training sessions as 12 or higher on the Borg Scale, which is in line with findings from a recent study showing that in PPS, the heart rate attained at the AT corresponds well to a score of 12 on the Borg Scale [24]. Nonetheless, we found no indications of increased cardiorespiratory fitness levels following the aerobic training program.

The hypothesis that our training program resulted in positive muscular adaptations, which, due to the reduced muscle mass of the lower extremities, did not lead to an increased cardiorespiratory fitness, seems unlikely. As for the cardiorespiratory fitness, we found no indications of an improved lower extremity muscle function–i.e. neither muscle endurance nor muscle strength of the knee extensor muscles significantly improved in the ET group, compared to UC. It is however important to realize that findings regarding muscle function, especially those for muscle endurance, should be interpreted with caution because they are based on a small number of observations. Possibly, the presence of muscular adaptations could not be detected due to the small sample size [28].

The absence of muscular adaptations in our study is consistent with findings from Willén and colleagues who also found no change in knee extensor muscle function following a 5-month dynamic water exercise program [8]. Contrary, Ernstoff and colleagues found an increased muscle strength in some–mainly upper extremity–muscle groups, as well as an increased fatigue resistance of the weaker leg, though without changes in aerobic enzyme activity or cross-sectional areas of the muscle fibers [7]. A possible explanation for the absence of muscular adaptations in our study is that the involved muscles were already adapted considerably in response to the relative higher loading during daily life activities [26,29]. This is supported by findings of extensive type I fiber predominance and hypertrophied muscle fibers in PPS [30,31]. Also a recent study showed no deconditioning of the knee extensor muscles as compared with healthy subjects [26]. It may therefore be difficult to improve muscular function and (accordingly) cardiorespiratory fitness through exercise that primarily focuses on muscles of the lower extremities. The increased cardiorespiratory fitness found by Willén, Ernstoff and colleagues [7,8] could be explained from the fact that their training programs aimed at whole body exercise.

Another finding from our study is that training dose was not associated with the change in cardiorespiratory fitness. This contrasts findings in runners and young soccer players, where explained variance values ranging from 45% to 76% were reported [32,33]. Possibly, this inconsistency relates to the different methods used for calculating the individual training dose. Had we used a more individualized approach, based on individual physiological responses, this might have resulted in a stronger association [32,33]. On the other hand, in healthy persons, the large variability in the way individuals react to training is, besides training dose, influenced by several other factors [27]. It is therefore conceivable that also in PPS, other, probably disease specific factors, play a role as well. For example, the extent to which the muscles have already been adapted will influence the way individuals with PPS react to training [30,31].

### Strengths and limitations

We carefully monitored the HR of individuals during their home-based training program, enabling us to quantify the actual training dose. The finding that, contrary to earlier reports most individuals with PPS did not adhere to a high intensity program emphasizes the necessity to monitor the actually achieved training dose and to reconsider the application of such programs in clinical practice. On the other hand, there was a pattern of increasing duration and intensity throughout the entire training program. While speculative, if the exercise program had been extended, the designated intensities might have been reached for some of the participants. Not selecting participants based on the presence of deconditioning could be considered another limitation of the present study. Possibly, a more targeted selection, aimed at participants with deconditioned muscles, would have yielded better treatment effects.

### Clinical implications

Even though it remains uncertain what the optimal training intensity for improving the aerobic capacity is, we now know that, for most individuals with PPS intensities of 60%HRR or more are not sustained during prolonged periods. Instead, training intensity prescription based on the AT or, alternatively, on ratings of perceived exertion, rather than on a fixed %HRR for the entire study group, offers a more individualized target for aerobic training in PPS [24]. Whether this can also be applied to other exercise modes such as arm ergometry or four-limb ergometry is uncertain and requires further investigation.

Increasing the aerobic capacity through exercise may be possible in PPS, provided that training programs are highly individualized with respect to the aerobic (muscle) capacity. In some individuals, the muscles that are used during activities in daily life probably have already been considerably adapted as a consequence of extensive use, limiting the potential for adaptations to these muscles. For those individuals, increasing the aerobic capacity might be possible by using exercise modes that require the use of other large muscle groups instead. In other individuals, where the muscles that are required for daily tasks are deconditioned due to disuse, training should include exercise modes that require the use of those particular muscle groups, in order to improve the aerobic capacity. Therefore, when prescribing aerobic exercise in PPS, one should determine whether functionally important muscle groups are disused during daily life activities. If this is the case, those muscle groups should be involved in the training program. If this is not the case, the use of other large muscle groups instead should be considered. One must realize, however, that if the involved muscles are not used during daily life activities, the obtained training effects will not sustain.

## Conclusion

Despite high attendance rates, severely fatigued individuals with PPS did not adhere to a 4-month high intensity home-based aerobic training program on a bicycle ergometer. Although participants instead trained around their AT most of the training period, the program did not result in an increased aerobic capacity, as muscle function nor cardiorespiratory fitness improved. Improving the aerobic capacity is difficult through exercise primarily focusing on the lower extremities, and requires a more individualized approach, including the use of other large muscle groups instead. A possible next step is to study the efficacy of training programs tailored to the individual’s aerobic (muscle) capacity, that may eventually alleviate fatigue in individuals with PPS.

## Supporting Information

S1 CONSORT Checklist (DOC)

S1 Protocol Exercise therapy and cognitive behavioural therapy to improve fatiue, daily activity performance and quality of life in Postpoliomyelitis Syndrome: the protocol of the FACTS-2-PPS trial. (PDF)
