# Correction: Aerobic Exercise Training in Post-Polio Syndrome: Process Evaluation of a Randomized Controlled Trial

**DOI:** 10.1371/journal.pone.0192338

**Published:** 2018-01-30

**Authors:** Eric L. Voorn, Fieke S. Koopman, Merel A. Brehm, Anita Beelen, Arnold de Haan, Karin H. L. Gerrits, Frans Nollet

There is an error in Table 1. The values in the “ET” column are swapped with values in the “UC” column. Please see the corrected Table 1 here.

**Table 1:** Baseline characteristics of participants.

	ET (N = 22)	UC (N = 22)
**Demographic data**		
Age (*yr*), mean (SD)	56.7 (8.9)	60.1 (7.4)
Gender, n female (*%*)	13 (59%)	11 (50%)
Body mass index (*kg/m*^*2*^), mean (SD)	25.6 (4.3)	26.5 (3.4)
**Polio characteristics**		
Age at acute polio (*yr*), median (range)	2 (0–16)	3 (1–40)
Time since new symptoms (*yr*), mean (SD)	14.6 (9.5)	13.2 (7.7)
Present walking distance, n(%)^a^ Around the house Seldom further than 1 km Regularly further than 1 km	6 (27%) 11 (50%) 5 (23%)	9 (41%) 8 (36%) 5 (23%)
Manual muscle testing sum score legs (*0 to 80*), median (range)^b^	69.6 (43.0–80.0)	67.8 (41.8–80.0)
Manual muscle testing sum score arms (*0 to 80*), median (range)^c^	50.0 (16.8–50.0)	50.0 (32.0–50.0)

Abbreviations: ET, exercise therapy; UC, usual care.

^a^ Walking distance was classified into 3 categories: around the house, seldom further than 1 km, and regularly further than 1 km.

^b^ Sum score for muscle strength of the legs was calculated by adding 16 muscle groups. Each muscle group had a score between 0 and 5, sum score ranged from 0 to 80 [19].

^c^ Sum score for muscle strength of the arms was calculated by adding 10 muscle groups. Each muscle group had a score between 0 and 5, sum score ranged from 0 to 50 [19].
